# Supplementary material for: Light-independent regulation of algal photoprotection by CO2 availability
Source: Nat Commun. 2023 Apr 8;14:1977. doi: 10.1038/s41467-023-37800-6 (PMC10082802; doi:10.1038/s41467-023-37800-6)
Supplement: Supplementary file 1 — Supplementary Information [file 41467_2023_37800_MOESM1_ESM.pdf]

Supplementary Information

This PDF file includes:

Supplementary Note 1
Supplementary Figs 1 to 8
Supplementary Tables 1 to 4

Other Supplementary Materials for this manuscript include the following:

Supplementary Datasets 1 to 3 (to be downloaded as excel file)

Supplementary Note 1

Application of flux balance analysis to investigate proxies for internal CO_2 concentration. We used flux balance analysis (FBA)[1] to test whether the genome-scale model of *Chlamydomonas* *i*Cre1355[2] could accurately predict the specific growth rates (generation time) of the *icl* and *dum11* strains under autotrophic LL, autotrophic HL and mixotrophic (acetate) HL conditions. We first constrained the specific growth rates of WT to the measured values under LL and HL[3] and determined the respective minimum photon uptake supporting the specific growth rates (Methods). Assuming that the photon uptake is not altered in the mutants, the determined minimum photon uptake was then used as a bound in the FBA-based prediction of generation times for the *icl* and *dum11* strains. In line with the experimentally observed values, we found that the predicted generation times for the *icl* and *dum11* strains grown autotrophically under LL did not differ from those of LL grown WT cells (**Supplementary Table 1**). In comparison to LL conditions, the predicted generation time of the *icl* and *dum11* strains grown autotrophically under HL decreased by 1.8- and 1.6-fold, respectively, which was also similar to that of WT cells (**Supplementary Table 1**). However, the predicted generation time for the WT grown under mixotrophic HL conditions further declined by 2-fold, to 11 h while the mutants under mixotrophic HL conditions had a generation time similar to that observed under autotrophic HL conditions (19 h, **Supplementary Table 1**). These findings showed that the FBA-based modeling with the condition-specific constraints can reproduce experimental findings regarding strain- and condition-specific generation times.

We next examined whether the CO_2-producing reactions show flux differences between the WT and the mutant strains under the specific growth conditions used. To this end, we followed two strategies, one based on the differences in the flux ranges and another based on the differences in the sampled steady-state flux distributions. By using the first strategy, we found a number of reactions whose steady-state flux ranges did not overlap between the WT and modelled mutants under the investigated conditions (**Supplementary Dataset 1**), suggesting clear redistribution of fluxes between WT and mutant. The second strategy allowed us to identify reactions that showed both significant differences in the distributions of the sampled flux values, using the Kolmogorov-Smirnov test, and significant difference in means between WT and the mutants, based on t-tests (**Supplementary Dataset 2**). The Fisher's exact test was in turn used to assess if the set of reactions showing differences are enriched with CO_2-producing reactions. Our findings showed that there is a significant difference (p-value = 0.003) in the flux of CO_2-producing reactions between WT and mutants for mixotrophic growth under HL conditions; analogous conclusions were made when only focusing on the CO_2-producing reactions in the chloroplast (p-value= 6.4e-04; **Supplementary Table 2**). However, this could not be observed under LL and HL photoautotrophic conditions. In addition, we ask if the observed difference in flux of CO_2-producing reactions for the mixotrophic HL condition is a result of (in)activation of certain reactions between WT and mutant. To test this hypothesis, we calculated the Jaccard distance between the sets of active CO_2-producing reactions. We found that the set of active reactions is the same for all strains indicated by a Jaccard distance of zero. Next, we aim to investigate if the change in flux for CO_2-producing reactions is due to an altered relative contribution of reaction fluxes to production of CO_2. Therefore, we compute flux splits[4] and find that the relative contribution cannot explain the difference in the phenotype (**Supplementary Table 3**). To further investigate how this observation may propagate to other pathways, i.e., model subsystems, we investigate which pathways show the largest differences between the mutants and the WT for

mixotrophic growth under HL. As a result, we calculate the percentage of reactions that show a significant difference in flux between WT and mutants only under mixotrophic HL condition (**Supplementary Dataset 3**). We found the highest percentage of reactions with significant change for mixotrophic HL condition, but no change under autotrophic LL and HL, for the following pathways: N-Glycan biosynthesis (100%, 26 reactions) and protein synthesis (100%, 1 reaction), followed by tyrosine metabolism (57%, 4 reactions) and valine, leucine and isoleucine degradation (52%, 17 reactions). In addition, pathways like fatty acid biosynthesis (47%, 29 reactions), nitrogen metabolism (45%, 10 reactions), photosynthesis (44%, 4 reactions) as well as starch and sucrose metabolism (40%, 6 reactions) and glycerolipid metabolism (40%, 138 reactions) fall in the highest ranked pathways with respect to percentage of reactions with significant change for mixotrophic HL. These observed changes in fluxes may be explained by transcriptional reprogramming that affect downstream enzyme abundances who support the flux changes. As demonstrated in our experimental validation, CO_2 can serve as a signal for these transcriptional reprogramming. In addition, other mechanisms related to allosteric regulation of reaction rates cannot be excluded.

Prompted by these findings, we interrogated whether or not changes in flux are associated with changes in the internal CO_2 concentration. Since FBA cannot be used to predict concentrations of metabolites, we used a technique employed in the design of metabolic engineering strategies to modulate the production (and hence concentration) of a metabolite of interest. This technique entails insertion of a synthetic ‘demand’ reaction for the metabolite of interest, which exports the metabolite out of the network. In our case, we inserted a demand reaction for CO_2 from the chloroplast to the environment, and used its maximum flux, at the specific condition associated with strain-specific-growth constraints, as a proxy for intracellular CO_2. We then inspected the condition-specific flux through the added demand reaction for different combinations of CO_2 and acetate uptake rates (**Supplementary Fig. 2**). We observed the same flux pattern for the CO_2 demand reaction with varying rates of CO_2 uptake from the environment across all strains under autotrophic LL and HL. We hypothesized that under LL more CO_2 can accumulate because of slow carbon fixation in comparison to HL conditions, where CO_2 fixation is faster. In support of this hypothesis, we found that all strains showed larger flux through the CO_2 demand reaction, as a proxy for the internal CO_2 levels, under LL than HL conditions when the CO_2 uptake rates were larger than 0.2 mmol/gDW/h (**Supplementary Fig. 2a-c**). Under mixotrophic HL conditions, with the assumption of no change in CO_2 uptake from the environment and a decrease of at least 10% in acetate uptake for both mutants in comparison to WT, we found that both the *icl* and *dum11* mutants showed smaller flux through the CO_2 demand reaction, i.e. lower internal CO_2 concentration than what was observed for WT (**Supplementary Fig. 2d-f**). Furthermore, the same pattern holds with the assumption that CO_2 uptake under HL is at least as high as under LL and acetate uptake rates are below 0.3 mmol/gDW/h. In contrast, only few combinations of CO_2 and acetate uptake rates for which the mutant strains showed CO_2 demand that is similar under autotrophic LL and mixotrophic HL conditions, but larger than the CO_2 demand in the WT under auxotrophic LL conditions. Therefore, we concluded that larger CO_2 demand flux under autotrophic LL than HL conditions for each strain can be observed with the assumptions that: (i) the CO_2 uptake was not affected by the mutation, (ii) CO_2 uptake is the same for phototrophic HL and mixotrophic HL, (iii) CO_2 uptake under HL is at least as high as under LL and (iv) the acetate uptake rate is low (i.e., below 0.3 mmol/gDW/h) for the mutants (as indicated in **Fig. 2c** and **f**). Moreover, under mixotrophic HL conditions, both mutants exhibited CO_2 demand rates that were smaller than those under autotrophic LL conditions. In contrast, the WT showed a marked increase

in the CO_2 demand flux under mixotrophic HL conditions in comparison to autotrophic LL and HL, indicating higher internal CO_2 concentrations in the presence of acetate. In conclusion, genome-scale metabolic modelling supports the hypothesis that there are changes in the internal CO_2 concentration under autotrophic and mixotrophic growth conditions at different light intensities. These changes are congruent with the changes in the accumulation of *LHCSR3* transcripts under the different media conditions and in the WT and mutant cells.

Condition and strain-specific metabolic models

Simulations of different strain, $s = \{WT, icl, dum11\}$, and conditions, $c = \{LL, HL, HL + \ acetate\}$, are based on the genome-scale metabolic network reconstruction *i*Cre1355 of *Chlamydomonas reinhardtii* metabolism [2]. The reconstruction provides the underlying structure of the metabolic reactions captured in the stoichiometric matrix, $\boldsymbol{N}$, where rows correspond to metabolites and columns denote reactions. Each entry in the stoichiometric matrix indicates the molarity with which a metabolite is consumed (negative value) or produced (positive value) by the respective reaction. In addition, condition-specific lower and upper bounds on reaction flux, $v_{min}^{WT,c} \leq v \leq v_{max}^{WT,c}$, for autotrophic (LL, HL) and mixotrophic (HL + acetate) growth are provided with the model. To obtain models for the mutants *icl* and *dum11* we used the gene-protein-reaction rules, provided along the network reconstruction, to identify reactions related to knocked-out genes Cre06.g282800 and CreMt.g000300, respectively. Gene Cre06.g282800 relates to reaction isocitrate lyase and therefore, flux through this reaction is blocked in the simulations of *icl*. For the mutant *dum11* the knocked-out gene CreMt.g000300 was not part of the model. However, it is known that this mutant shows no activity of respiratory complex III, therefore the corresponding model reaction was blocked in the simulation of *dum11*.

The strain and condition-specific simulations, together with constraint-based modeling approaches were used to investigate steady state flux distributions, v. First, we used the WT model under autotrophic conditions to obtain estimates for photon uptake rates under LL and HL conditions, later used as constraints in the mutant models and for the WT model under HL + acetate condition. Therefore, we take generation time (g) of *Chlamydomonas* WT under LL and HL measured by Bonente et al. [3] and converted them into growth rates (μ) assuming that $g = \frac{log(2)}{\mu}$. The respective growth rate was used to constrain the WT model under LL and HL conditions. To estimate photon uptake in units mmol gDW^{-1} h^{-1} under low and high light conditions, we found the minimum photon uptake rate that supports the condition-specific WT growth rate (bio_c^{WT}) under LL and HL, respectively (Eq. 1). The resulting photon uptake rates were used as constraints for the simulation of mutants as well as under HL mixotrophic growth for the WT (were no measured growth rates were available). The following is the linear program that we solve:

$$
\begin{aligned}
& z_{photon_{uptake}}^{c} = \min v_{photon_{uptake}} \\
& s.t. \\
& Nv = 0 \qquad\qquad (1) \\
& v_{bio} = bio_c^{WT} \\
& v_{min}^{WT,c} \leq v \leq v_{max}^{WT,c}.
\end{aligned}
$$

Next, we used the observation that mutants cannot grow on acetate in darkness to find acetate uptake rates that allow simulation of no growth in darkness for both mutants. Acetate uptake for both mutants were reduced by 90% in comparison to the WT rate (0.2 mmol gDW^{-1} h^{-1} in mutants and 2 mmol gDW^{-1} h^{-1} in WT), since this rate is the minimum uptake rate for which no growth was simulated in darkness.

Flux balance analysis

To simulate maximal growth rates for WT under HL + acetate as well as *icl* and *dum11* under LL, HL and HL + acetate respectively, we applied flux balance analysis (FBA; [5,6]) We found maximal growth rates (Eq. 2) by using the model biomass reaction for mixotrophic and photoautotrophic growth and the respective light constraints. Moreover, acetate uptake for mutant models under HL + acetate was set to 0.2 mmol gDW^{-1} h^{-1}, the minimum acetate uptake rate for which no growth was simulated in darkness. To this end, we used the following program:

$$\begin{aligned} &z_{bio}^{s,c} = \max v_{bio} \\ &s.t. \\ &Nv = 0 \\ &v_{photon_{uptake}} = z_{photon_{uptake}}^{c} \\ &v_{min}^{s,c} \leq v \leq v_{max}^{s,c}. \end{aligned} \qquad (2)$$

Flux ranges

The solution of the linear programming problem in Eq. (2), above, is the maximum growth, i.e. flux value of the strain and condition-specific biomass reaction, $z_{bio}^{s,c}$. Flux variability analysis (FVA) allows determining the minimum and the maximum value of flux that a given reaction can carry while ensuring maximum flux through the biomass reaction[7]. These values can be obtained by solving the following linear program for a given reaction i. The flux through the condition-specific biomass reaction was set to 99% of the optimum to avoid numerical instabilities. To conduct FVA, we solved the following linear programs:

$$\begin{aligned} &z_{i,\max(\min)}^{s,c} = \max(\min)\ v_i \\ &\text{s.t.} \\ &Nv = 0 \\ &v_{photon_{uptake}} = z_{photon_{uptake}}^{c} \\ &v_{min}^{s,c} \leq v \leq v_{max}^{s,c} \\ &v_{bio} = z_{bio}^{s,c} * 0.99 \end{aligned} \qquad (3)$$

Moreover, we sample 5000 feasible steady-state flux distributions from the flux cone of the strain and condition-specific models by applying the function *gpSampler* from the COBRA toolbox[8]. Here, too, biomass was set to 99% of its optimum.

Non-overlapping flux ranges

Flux ranges between wild type and mutants are considered to not overlap for condition c if (1) $z_{i,\max}^{WT,c} < z_{i,\min}^{\{icl,dum11\},c}$ or $z_{i,\max}^{\{icl,dum11\},c} < z_{i,\min}^{WT,c}$, i.e. the minimum flux obtained from FVA (see Eq. 3) in a mutant is larger than the maximum flux obtained for the wild type or the minimum flux in the wild type is larger than the maximum flux for the mutant; i.e. there is no intersection between the flux ranges; (2) minimum flux for WT and mutants is greater than 0.01 mmol gDW^{-1} h^{-1}, to avoid considering reactions with low absolute flux; and (3) in line with differential expression analysis, where one considers genes differentially expressed above a preselected fold-change (e.g. of at least 2, in nominal values), for the flux ranges that do not overlap, we use a threshold on the relative difference between the lower bounds of at least 5% (we used 5% to be less restrictive) to filter for cases were flux ranges are close to each other; this condition is meant to remove any numerical artifacts.

Maximize CO_2 demand

We introduce a demand reaction for CO_2 in the chloroplast and maximize its flux given constraints described in the linear program in Eq. (4), which include the fixation of condition-specific growth as well as uptake rates of CO_2 and acetate. The obtained flux through CO_2 demand will serve as a proxy for internal CO_2 concentration in the chloroplast:

$$
\begin{aligned}
& z^c = \max\ v_{CO_2\ demand} \\
& \text{s.t.} \\
& Nv = 0 \\
& v_{photon_{uptake}} = z^c_{photon_{uptake}} \\
& v_{CO_2\ uptake} = CO_2\ uptake^c \\
& v_{acetate\ uptake} = acetate\ uptake^c \\
& v^{s,c}_{min} \le v \le v^{s,c}_{max} \\
& v_{bio} = z^{s,c}_{bio} * 0.99
\end{aligned}
\qquad (4)
$$

Supplementary References

1. Varma, A. & Palsson, B. O. Metabolic capabilities of Escherichia coli: I. synthesis of biosynthetic precursors and cofactors. *J. Theor. Biol.* **165,** 477–502 (1993).
2. Imam, S. *et al.* A refined genome-scale reconstruction of Chlamydomonasmetabolism provides a platform for systems-level analyses. *Plant J.* **84,** 1239–1256 (2015).
3. Bonente, G., Pippa, S., Castellano, S., Bassi, R. & Ballottari, M. Acclimation of Chlamydomonas reinhardtii to different growth irradiances. *J. Biol. Chem.* (2011). doi:10.1074/jbc.M111.304279
4. Aurich, M. K., Fleming, R. M. T. & Thiele, I. MetaboTools: A Comprehensive Toolbox for Analysis of Genome-Scale Metabolic Models. *Front Physiol* **7,** 327 (2016).
5. Orth, J. D., Thiele, I. & Palsson, B. Ø. What is flux balance analysis? *Nat. Biotechnol.* **28,** 245–248 (2010).
6. Bordbar, A., Monk, J. M., King, Z. A. & Palsson, B. Ø. Constraint-based models predict metabolic and associated cellular functions. *Nat. Rev. Genet.* **15,** 107–120 (2014).
7. Gudmundsson, S. & Thiele, I. Computationally efficient flux variability analysis. *BMC Bioinformatics* **11,** 489–3 (2010).
8. Heirendt, L. *et al.* Creation and analysis of biochemical constraint-based models using the COBRA Toolbox v.3.0. *Nature Protocols* **14,** 639–702 (2019).

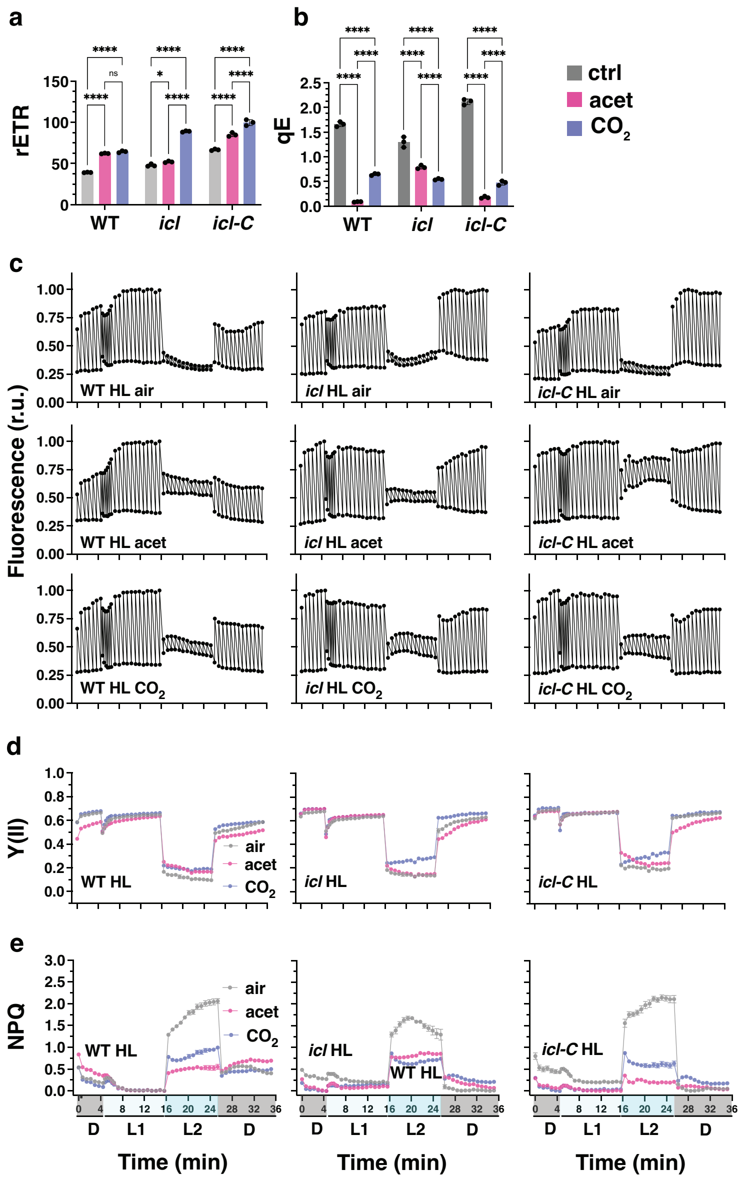

Supplementary Fig. 1: Effect of carbon availability on the photosynthetic properties of WT, *icl* and *icl-C* cells. a relative photosynthetic electron transfer rETR measured at 336 µmol photons m^{-2} s^{-1} and **b** qE of WT, *icl* and *icl-C* cells exposed to 600 µmol photons m^{-2} s^{-1} in HSM for 4h; sparged with air (labelled as "air"); sparged with air and supplemented with 10 mM sodium acetate (labelled as "acet); sparged with air enriched with 5% CO_2 (labelled as "CO_2"), (*n* = 3 biological samples, mean ± s.d.). The statistical analyses (two-way ANOVA with Tukey's multiple comparisons tests) of **a** and **b** are shown in the graph; * = *P* value < 0.05, ***=P value <0.001. Exact p-values can be found at the Source Data file. **c** Raw data of *in vivo* chlorophyll fluorescence (normalized to the highest Fm') for WT, *icl* and *icl-C*. Chlorophyll fluorescence was recorded in the dark (labelled as "D"), at 21 (labelled as "L1") and 336 (labelled as "L2") µmol photons m^{-2} s^{-1} as indicated in the graphs. Shown are one representative trace of three biological replicates. **d** Y(II) values calculated as (Fm'-F)/Fm' (*n* = 3 biological samples, mean ± s.d.). **e** NPQ values calculated as (Fm − Fm′)/Fm (*n* = 3 biological samples, mean ± s.d.). Please not that in **d** and **e** most of the error bars are smaller than the data points and are therefore not visible.

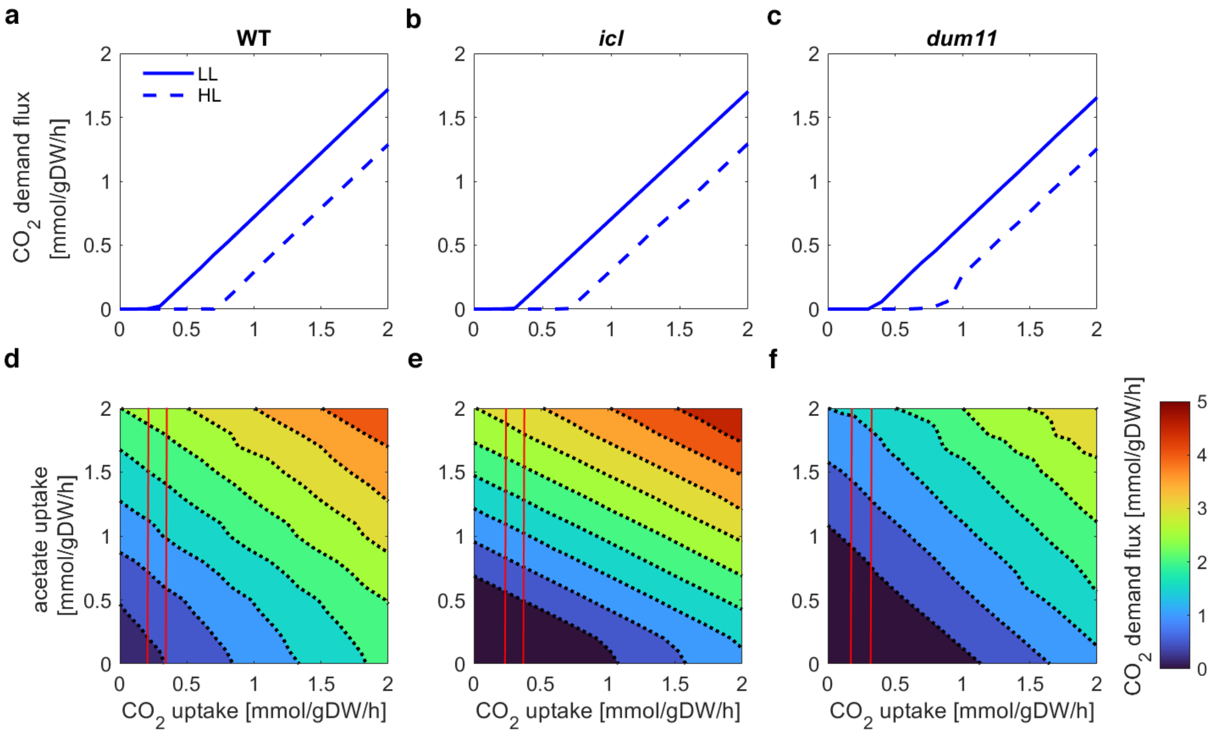

Supplementary Fig. 2: Large-scale metabolic modeling supports the change in internal CO_2 concentration under mixotrophic conditions. The maximum flux through the CO_2 demand reaction is used as a proxy for concentration of free CO_2 in the chloroplast. **a-c** Flux through CO_2 demand reaction under low light (LL) and high light (HL) for varying CO_2 uptake rates for wild type (wt), *icl*, and *dum11*. **d-f** Contour plots of flux through CO_2 demand reaction under HL and acetate for varying CO_2 and acetate uptake rates for the three respective strains. The area between the two red lines indicates combinations of CO_2 and acetate uptake rates that show (i) in the case of mutants: CO_2 demand level under HL acetate conditions similar to those under LL conditions and (ii) in the case of the WT: CO_2 demand level under HL acetate conditions above what is observed under LL conditions.

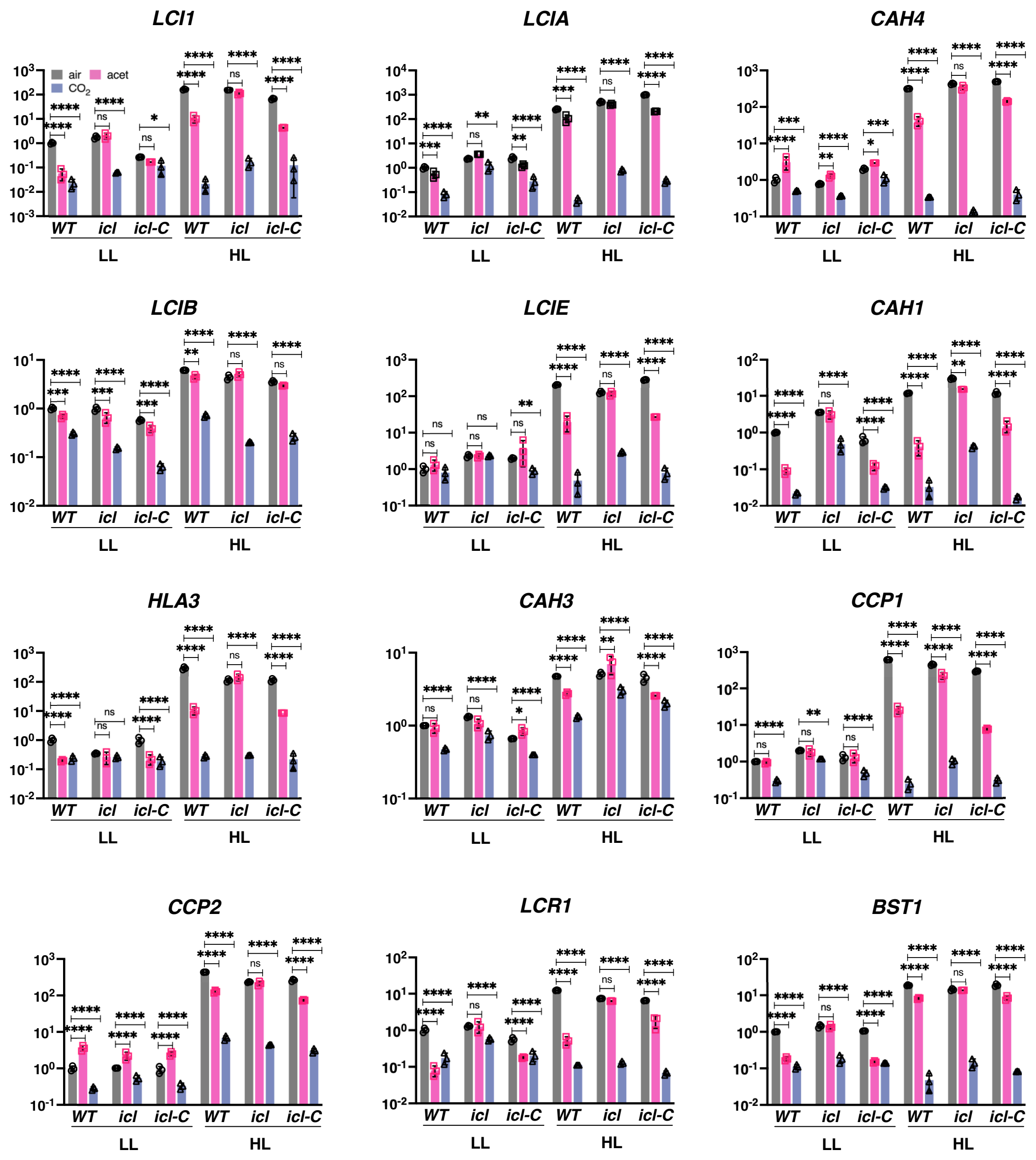

Supplementary Fig. 3: HL and low-CO_2 responses cross-talk. WT, *icl* and *icl-C* strains were acclimated for 16 h in LL (15 µmol photons m^{-2} s^{-1}) in HSM; bubbled with air (labelled "ctrl"); bubbled with air and supplemented with 10 mM sodium acetate (labelled "acet); bubbled with air enriched with 5% CO_2 (labelled "CO_2"). After sampling for the LL conditions, light intensity was increased to 600 µmol photons m^{-2} s^{-1} (HL); samples were taken after 1h. Accumulation of mRNA of selected CCM genes at the indicated conditions normalized to WT LL ctrl. Please note that these data derive from analyses of the RNA samples of the experiment described in **Fig. 1** (n = 3 biological samples, mean ± s.d.). The p-values for the comparisons of acetate and CO_2 conditions to air are based on ANOVA Dunnett's multiple comparisons test of log10 transformed mRNA data as indicated in the graphs (*, $P < 0.005$, **, $P < 0.01$, ***, $P < 0.001$, ****, $P < 0.0001$, ns, not significant). Exact p-values can be found at the Source Data file.

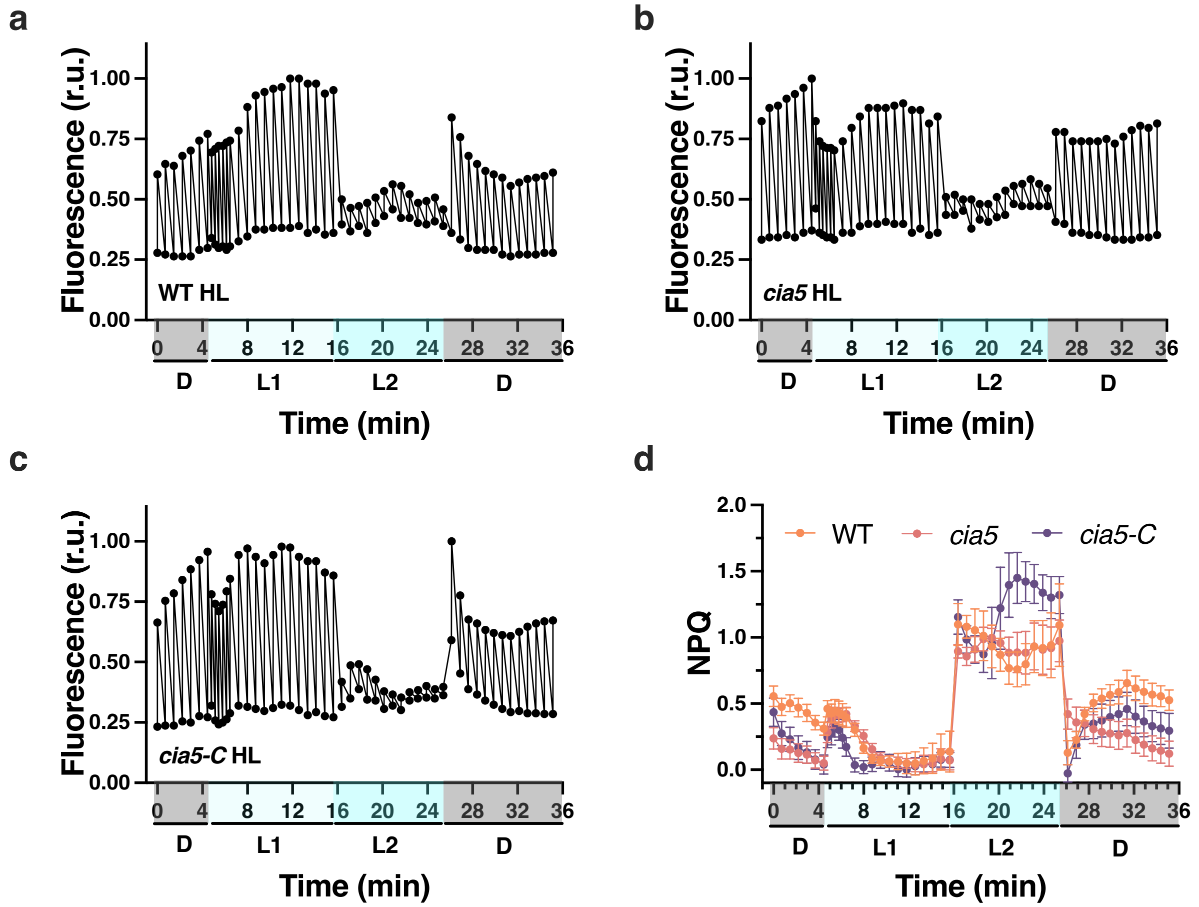

Suppementary Fig. 4: Detailed view of photosynthetic measurements of Fig. 3c.
WT, *cia5* and *cia5-C* strains were acclimated for 16 h in LL (15 µmol photons m^{-2} s^{-1}) in HSM bubbled with air (labelled as "LL"); after sampling for the LL conditions, light intensity was increased to 600 µmol photons m^{-2} s^{-1} (HL); samples were taken after 4 h for photosynthesis measurements). **a-c** *In vivo* chlorophyll fluorescence (normalized to the highest Fm') of HL-acclimated WT, *cia5* and *cia5-C* cells (for a complete description of the experimental setup please refer to the legend of **Fig. 4**.) Just prior to the onset of the measurements, cells were acclimated to darkness for 15 min. Chlorophyll fluorescence was recorded in the dark (labelled as "D"), at 21 (labelled as "L1") and 336 (labelled as "L2") µmol photons m^{-2} s^{-1} as indicated in the graphs. **d** NPQ values calculated as $(Fm - Fm')/Fm'$ ($n = 3$ biological samples, mean ± s.d.).

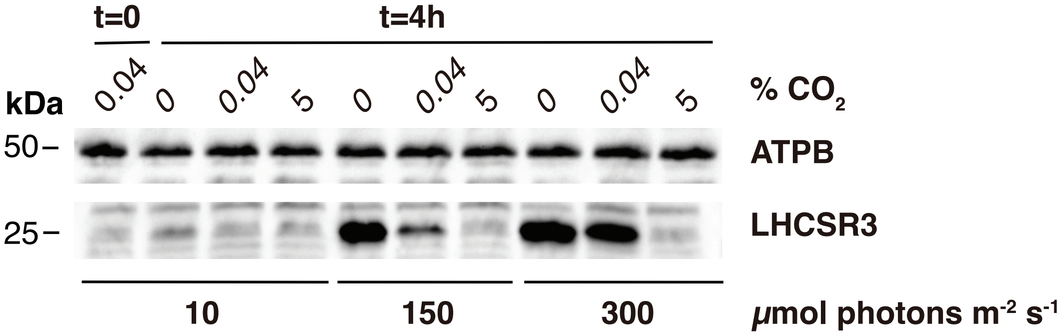

Supplementary Fig. 5: Light and CO_2 availability define expression levels of LHCSR3. WT cells, acclimated in LL (10 µmol photons m^{-2} s^{-1}) sparged with air were shifted to 10, 150, and 300 µmol photons m^{-2} s^{-1} of light and were sparged with 0 (100% O_2), 0.04 (air), and 5% CO_2 (95% O_2) for 4 hours. Shown are the immunoblot analyses of LHCSR3 and ATPB (loading control) under the indicated conditions. Representative dataset of experiment repeated three times.

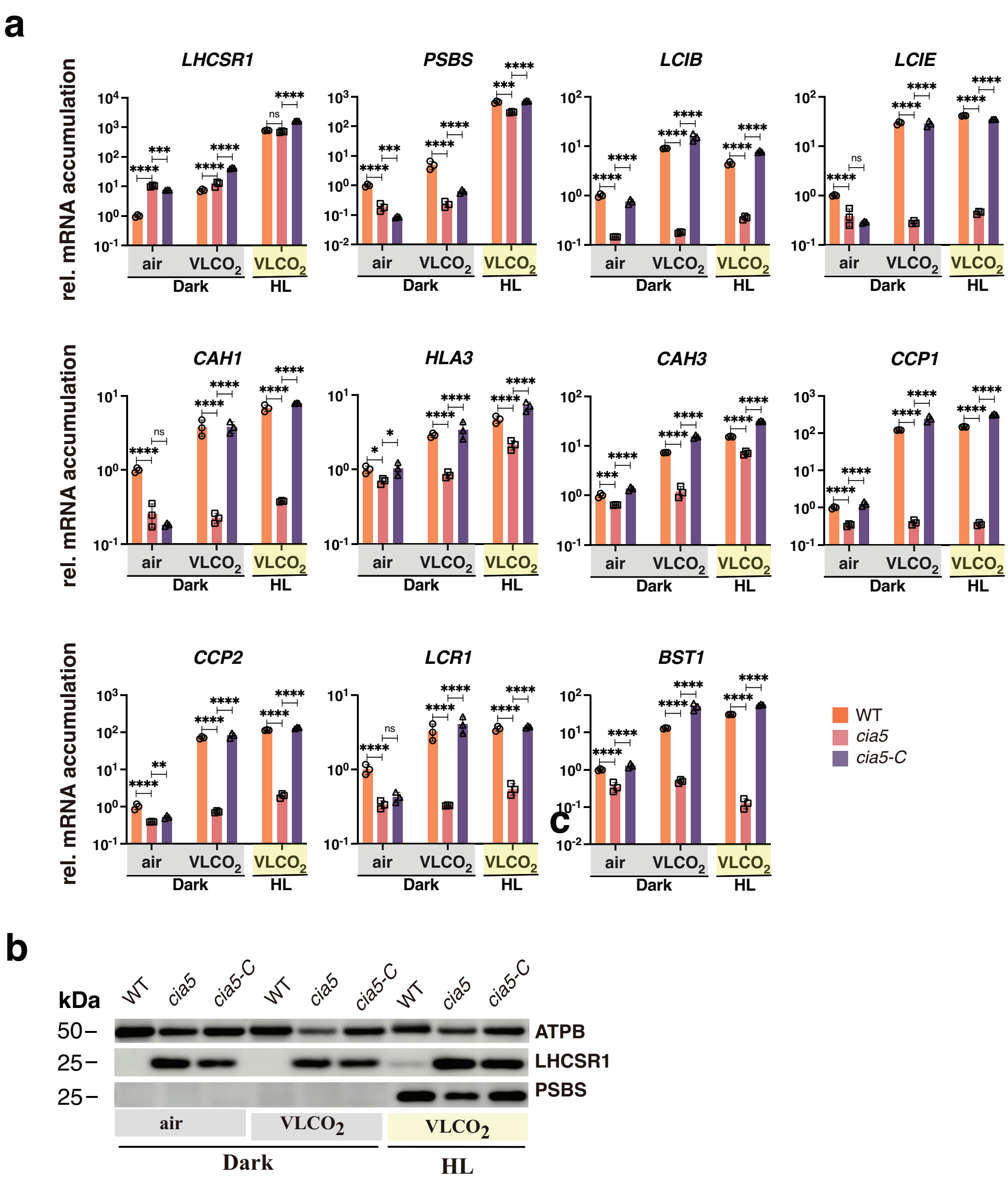

Supplementary Fig. 6: Low CO_2 levels can trigger CCM genes in the absence of light. WT, *cia5* and *cia5-C* cells were bubbled with air overnight in darkness; next day air bubbling was either maintained or replaced by CO_2-limited-air bubbling in the darkness or in the presence of 600 µmol photons m^{-2} s^{-1} light. Sampling was performed after 1 h (RNA) or 4 h (protein). **a** mRNA accumulation of *LHCSR1* and *PSBS1* (qE genes) and *LCIB, LCIE, CAH1, HLA3, CAH3, CCP1, CCP2, LCR1, BST1* (CCM genes) in WT, *cia5* and *cia5-C*. Data were normalized to WT air dark; (n = 3 biological samples, mean ± s.d.). The p-values for the comparisons of WT with *cia5* and *cia5* with *cia5-C* are based on ANOVA Dunnett's multiple comparisons test of log10 transformed mRNA data as indicated in the graphs (*, P < 0.005, **, P < 0.01, ***, P < 0.001, ****, P < 0.0001, ns, not significant). Exact p-values can be found at the Source Data file. **b** Immunoblot analyses of LHCSR1, PSBS and ATPB (loading control) under the indicated conditions.

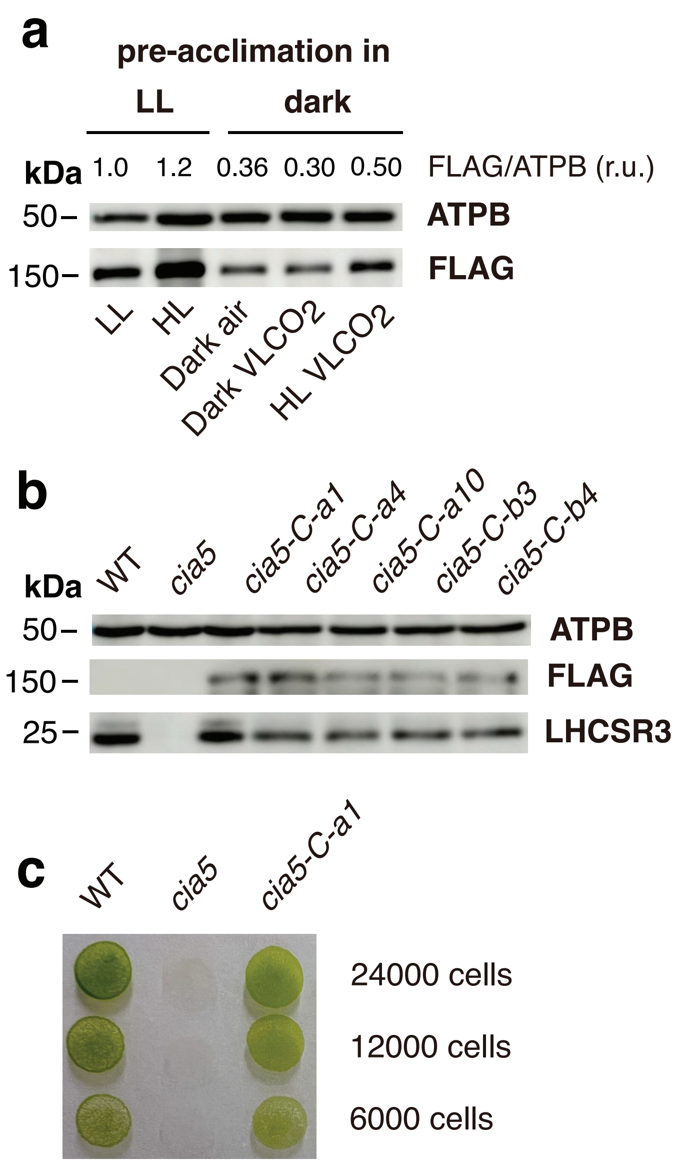

Supplementary Fig. 7: Complementation of *cia5* mutant. a Immunoblot analyses of CIA5-FLAG and ATPB (loading control) from whole cell extracts of *cia5-C*. The first two lanes, were loaded with *cia5-C* samples from the experiment presented in **Fig. 3b** (pre-acclimated in LL); the last three lanes contain *cia5-C* samples from the experiment presented in **Fig. 5b** (pre-acclimated in the dark). Above the immunoblot shown is the quantification of CIA5-FLAG protein accumulation (calculated as FLAG /ATPB ratio). Representative dataset of experiment repeated three times. **b** Immunoblot analyses of LHCSR3, CIA5-FLAG and ATPB (loading control) from whole cell extracts of WT, *cia5* and four *cia5-C* complemented lines after exposure at 300 µmol photons m^{-2} s^{-1} for 4 hours. Among the transformants analyzed the *cia5-C-a1* (*cia5-C* throughout the text) was retained for further analyses in the present study. Representative dataset of experiment repeated three times. **c** A total of 24, 12 and 6 x 10^3 cells of WT, *cia5* and *cia5-C-a1* were spotted on high-salt media agar plates and grown under 100 µE m^{-2} s^{-1} for four days.

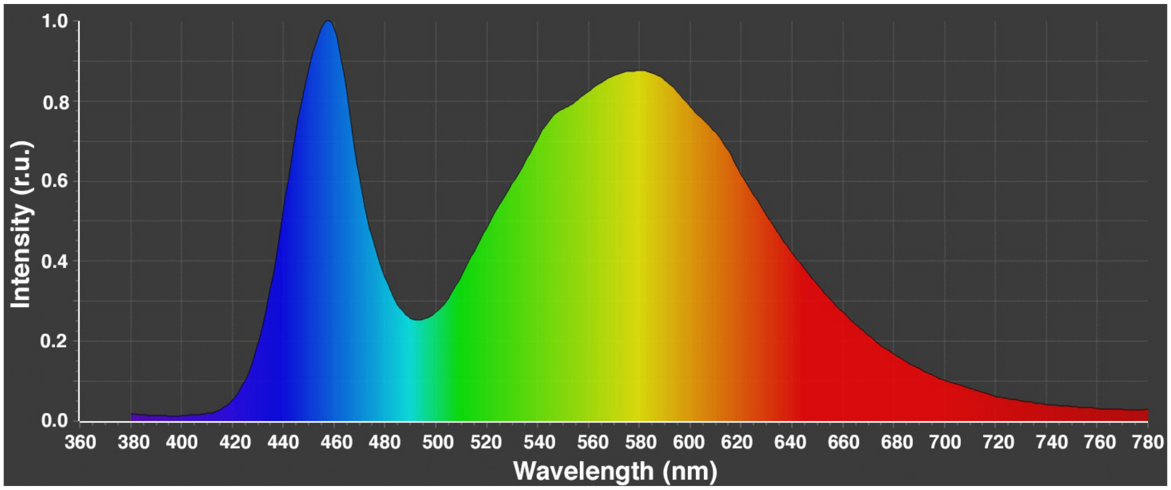

Supplementary Figure 8: Light Spectrum of the LED light system (Neptune L.E.D., France) used in the present study.

Supplementary Table 1. Predicted and observed generation time (h) for wild type and mutant strains under different growth conditions. Wild type and mutant strains were grown photoautotrophically under low light (LL) and high light (HL) conditions and cells grown mixotrophically on HSM medium were supplemented with acetate under high light (HL + acetate) conditions. Values followed by an asterisk represent constraints based on measurements, the rest of the values were simulated.

	**LL**	**HL**	**HL + acetate**
**WT**	36*	20*	11
icl	36	20	19
dum11	36	22	19

Supplementary Table 2. Overview number of reactions producing CO_2 with significant changes in flux between mutants (*icl* and *dum11*) and WT. (A) Across all model reactions; (B-D) compartment-specific reactions producing CO_2 - (B) cytosol, (C) chloroplast, (D) mitochondria. A significant enrichment in reactions with flux differences between mutants and WT for reactions that produce CO_2 under HL + acetate conditions (based on two-sided Fisher exact test) only was observed considering reactions in the chloroplast and considering all model.

A – all model reactions

	CO_2 producing	not CO_2 producing	p-value
**LL condition**			
change in flux	24	499	0.13
no change in flux	64	1807	
**HL condition**			
change in flux	19	504	0.57
no change in flux	69	1802	
**HL + acetate condition**			
change in flux	59	1186	**0.003**
no change in flux	29	1120	

B - cytosol

	CO_2 producing	not CO_2 producing	p-value
**LL condition**			
change in flux	7	313	0.48
no change in flux	24	1192	
**HL condition**			
change in flux	7	318	0.49
no change in flux	24	1187	
**HL + acetate condition**			
change in flux	18	840	0.48
no change in flux	13	665	

C - chloroplast

	CO_2 producing	not CO_2 producing	p-value
**LL condition**			
change in flux	9	71	0.37
no change in flux	21	206	
**HL condition**			
change in flux	8	68	0.48
no change in flux	22	209	
**HL + acetate condition**			
change in flux	24	133	**6.4e-04**
no change in flux	6	144	

D - mitochondria

	CO_2 producing	not CO_2 producing	p-value
**LL condition**			
change in flux	8	80	0.45
no change in flux	17	198	
**HL condition**			

change in flux	4	70	0.9
no change in flux	21	208	
**HL + acetate condition**			
change in flux	17	138	0.06
no change in flux	8	140	

Supplementary Table 3. Relative contribution of reaction flux to production of CO_2. The set of reactions with non-zero contribution is the same for all strains and conditions.

	LL			HL			HL+ac		
Reaction ID	WT	icl	*dum11*	WT	*icl*	*dum11*	WT	*icl*	*dum11*
'HCO3Em'	0.42	0.25	0.34	0.25	0.25	0.27	0.34	0.34	0.35
'ICDH_nad'	0.38	0.49	0.29	0.49	0.50	0.45	0.28	0.29	0.28
'GLYDHD'	0.19	0.25	0.35	0.25	0.24	0.27	0.35	0.35	0.35
'PPCKm'	0.00	0.01	0.01	0.01	0.01	0.00	0.01	0.01	0.01
'HCO3Ehi'	0.00	0.01	0.01	0.01	0.01	0.01	0.01	0.01	0.01

Supplementary Table 4. RT-qPCR primers for the genes analyzed in this study

Gene ID	Gene Name	Sense Primer	Anti-sense Primer
**Cre01.g016600**	PSBS1	TAAACCGTGTATTGGAACTCCG	CTCTGCACGCGGCGTGTT
**Cre08.g367500**	LHCSR3.1	CACAACACCTTGATGCGAGATG	CCGTGTCTTGTCAGTCCCTG
**Cre08.g365900**	LHCSR1	GAGTCTGAGATCACCCACGG	CCGATCTGCTGGAAGTGGTA
**Cre06.g278222**	GBLP	TGGCTTTCTCGGTGGACAAC	CTCGCCAATGGTGTACTTGC
**Cre06.g309000**	LCIA	AGATTTGATAACGGCAGGACC	CCTATCCCATGTCATTCCCAC
**Cre10.g452800**	LCIB	TGCATAAGAGCGGATGTAGC	CGGTAGTCAGCATCAGTCATC
**Cre04.g223250**	LCIE	TGCCGCCATAGATGTTGTGT	CCGCTCTTCTCTTTCGCTCA
**Cre09.g399552**	LCR1	GCACCAGCATACACCAAAATC	CAGAAAACAGAACGACCAAAG C
**Cre03.g162800**	LCI1	TTGCGGTTTTTGTACGAGCG	GTGCAAAGCCACGTCATCTC
**Cre02.g097800**	HLA3	CAGTGGCATGTTCCCTTTTG	GGTGCTCATGGTTCTTGTTTG
**Cre03.g162800**	CAH1	GCTTTGCTTCACGGTTTGGT	CCGGTACTGTGTGTATGCGT
**Cre09.g415700**	CAH3	AACCTGGAAGGGTGTGTGTG	CACTTCTCGAAGCTGCCGTA
**Cre05.g248400**	CAH4	CGAAAAGCTGCATGAACTCACC	GCCCGTAGGCTACAGTTTTC
**Cre04.g223300**	CCP1	TGGCATGACAACATGGCTCA	AGTGCATCCACTGGCTTGTT
**Cre04.g222750**	CCP2	AACGTGGAGCACATCTACGG	ATAAGCCGTCAAGCCTTGCT
**Cre16.g662600**	BST1	GCTGTGTGGCATTGAGGAGA	GGATGAGGCTGATGAGTCCG
**Cre06.g284100**	RHP1	GCAGTCGCAGGCAGTAACTA	CGCTTCAGCGCTCATAGAGA
**gib-cia5**		gctactcacaacaagcccagttATGGAAGCCTTAGACGC GC	gagccacccagatctccgttATCGCAGGA CTGCAGCAG

Supplementary Dataset 1 (available as downloadable Microsoft Excel file). Reactions whose minimum flux in the mutant was above the maximum flux of the WT (up-regulation) or the maximum flux in the mutant was below the minimum observed in the WT (down-regulation). Marked cells for down regulation under HL + acetate represent reactions also down regulated under LL in *dum11*.

Supplementary Dataset 2 (available as downloadable Microsoft Excel file). Flux ranges obtained from flux variability analysis as well as mean and median flux from sampling of 5000 flux distributions. Reactions marked in green show significant difference under HL+acetate but not under LL and HL conditions.

Supplementary Dataset 3 (available as downloadable Microsoft Excel file). Percentage of reactions per model pathway that show significant change in sampled flux values in both mutants, *icl* and *dum11* for the respective condition.
