# Supplementary material for: Light-independent regulation of algal photoprotection by CO2 availability
Source: Nat Commun. 2023 Apr 8;14:1977. doi: 10.1038/s41467-023-37800-6 (PMC10082802; doi:10.1038/s41467-023-37800-6)
Supplement: Supplementary file 3 — Description of Additional Supplementary Files [file 41467_2023_37800_MOESM3_ESM.pdf]

Description of Additional Supplementary Files:

Supplementary Dataset 1: (available as downloadable Microsoft Excel file). Reactions whose minimum flux in the mutant was above the maximum flux of the WT (up-regulation) or the maximum flux in the mutant was below the minimum observed in the WT (down-regulation). Marked cells for down regulation under HL + acetate represent reactions also down regulated under LL in dum11.

Supplementary Dataset 2: (available as downloadable Microsoft Excel file). Flux ranges obtained from flux variability analysis as well as mean and median flux from sampling of 5000 flux distributions. Reactions marked in green show significant difference under HL+acetate but not under LL and HL conditions.

Supplementary Dataset 3: (available as downloadable Microsoft Excel file). Percentage of reactions per model pathway that show significant change in sampled flux values in both mutants, icl and dum11 for the respective condition.
